# Allosteric antagonist action at triheteromeric NMDA receptors

**DOI:** 10.1016/j.neuropharm.2021.108861

**Published:** 2022-01-01

**Authors:** Alasdair J. Gibb

**Affiliations:** Research Department of Neuroscience, Physiology & Pharmacology, University College London, London, UK

**Keywords:** NMDA receptor, Triheteromeric receptor, Allosteric antagonist

## Abstract

NMDA receptors are one subtype of glutamate receptor that play fundamental roles in synaptic physiology and synaptic plasticity in the nervous system, in addition to being implicated in several neurological disorders. It is now established that many NMDA receptors in the nervous system are triheteromeric, composed of two glycine-binding GluN1 subunits and two different glutamate binding GluN2 subunits. The pharmacology of NMDA receptor has become well established since the pioneering work of Watkins and Evans almost half a century ago and has seen a resurgence of interest in the past decade as new subtype-selective allosteric modulators have been discovered. In this article, features specific to allosteric antagonist action at triheteromeric NMDA receptors are reviewed with a focus on understanding the mechanism of action of drugs acting at triheteromeric GluN1/GluN2B/GluN2D receptors. These receptors are of importance in the basal ganglia and in interneurons of the hippocampus and implications for understanding the action of allosteric antagonists at synaptic triheteromeric receptors are considered.

## Highlights

• Allosteric antagonist action at triheteromeric NMDA receptors are reviewed exemplified by GluN1/GluN2B/GluN2D receptors.
• A subunit-specific model of triheteromeric NMDA receptor activation is used to simulate allosteric antagonist action.
• Implications for understanding allosteric antagonist action at synaptic triheteromeric receptors are considered.

## 1 Introduction

### 1.1 Triheteromeric NMDA receptors

It is now more than 25 years since a landmark paper from Morgan Sheng and co-workers described biochemical evidence for NMDA receptors with two different GluN2 subunits within the same NMDA receptor (Sheng et al., 1994). It was an exciting period when understanding of the molecular composition of NMDA receptors was rapidly developing (Hollmann and Heinemann, 1994). Further biochemical evidence for triheteromeric NMDA receptors appeared in subsequent years (Chazot and Stephenson, 1997; Dunah et al., 1998) but it was more than a decade later before the importance of triheteromeric NMDA receptors in the CNS became established (Rauner and Köhr, 2011; Gray et al., 2011; Tovar et al., 2013; Bhattacharya et al., 2018) and the significance of this concept for NMDA receptor pharmacology began to be appreciated (Hatton and Paoletti, 2005; Hansen et al., 2014; Yi et al., 2019).

Concomitant with growing understanding of the molecular nature of NMDA receptors came the discovery of examples of subtype-selective antagonists that were non-competitive for the glutamate or the glycine binding sites exemplified by the GluN2B selective drugs such as ifenprodil (Williams, 1993), the Roche compound Ro25-6981 (Fischer et al., 1997) and the Pfizer compound CP101,606 (Mott et al., 1998). These allosteric antagonists have increased complexity in their mechanism of action compared to competitive antagonists (Traynelis et al., 2010; Paoletti, 2011; Tajima et al., 2016) as might be expected given the rich potential for domain and subunit drug interactions in a protein of the complexity of the NMDA receptor.

**Fig. 1:**
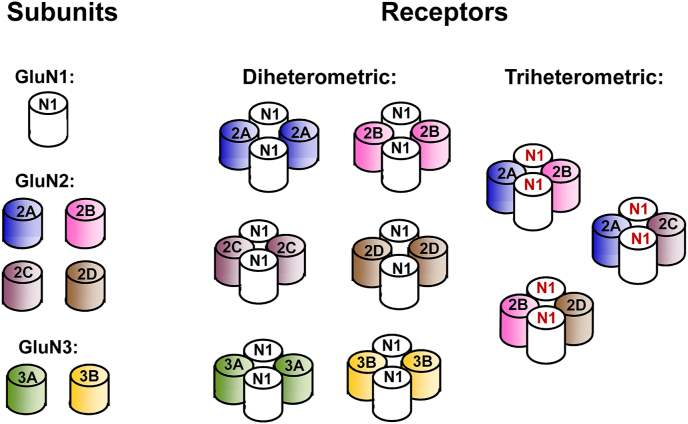
Example subunit combinations of NMDA receptor subunits making up diheteromeric and triheteromeric receptors containing two different GluN2 subunits. In addition, not illustrated here are the multiple GluN1 subunit splice variants that create the potential for further receptor diversity.

Our current molecular picture is that NMDA receptors are heterotetrameric complexes with most having two glycine-binding GluN1 subunits and two glutamate-binding GluN2 subunits (or a glycine-binding GluN3 subunit) arranged as a pair of heterodimers in a 1-2-1-2 order around the ion channel (Sobolevsky et al., 2009; Salussolia et al., 2011; Riou et al., 2012; Karakas and Furukawa, 2014; Lee et al., 2014) as illustrated in Fig. 1. Eight different GluN1 subunits are possible due to alternative splicing of the GluN1 subunit mRNA. Triheteromeric receptors have either two different glutamate-binding subunits or two different glycine-binding subunits. GluN3 containing triheteromeric receptors (Pérez-Otaño et al., 2016) and triheteromeric receptors which have two different GluN1 subunits will not be considered further here (Yi et al., 2018). In principle, tetraheteromeric NMDA receptors are also possible as a receptor could contain two different GluN1 subunits and two different GluN2 subunits (e.g. a GluN1-1a and GluN1-1b in a receptor with a GluN2A and GluN2B subunit). This paper will focus mainly on triheteromeric receptors containing GluN1/GluN2B and GluN2D subunits.

The NMDA receptor can be viewed as an allosteric protein complex which uses long-range interactions between discrete domains of the protein subunits to influence the stability of the open-channel conformation of the protein. The four subunits each contribute three membrane crossing alpha-helices and a re-entrant loop that come together to form the ion channel within a single receptor transmembrane domain (TMD); a cation selective pore that binds important non-competitive antagonists such as the channel blockers ketamine and memantine (Johnson et al., 2015). The structure of the intracellular, C-terminal domains of the subunits are less well defined but include important sites of phosphorylation and protein interaction. Each subunit also has two well-defined extracellular regions that have the structure of bilobed clamshells. The agonist binding domain (ABD) of the GluN2 subunit binds glutamate, while in the GluN1 subunit, this binds glycine or d-serine. The conformational change triggered by glutamate and glycine binding within the ABDs in each subunit allosterically controls channel open probability via conformational changes that are transmitted through the protein to the TMD. In this sense, glutamate and glycine can be viewed as the primary positive allosteric NMDA receptor modulators. The receptor subunits are arranged 1-2-1-2 and the bilobed agonist binding domains dimerize within a GluN1-GluN2 subunit pair contributing to the concept of the receptor as a ‘dimer of dimers'. Within each subunit, a second clamshell distal to the ABD and within the N-terminal domain (NTD), forms a regulatory domain controlling subunit assembly and channel open probability (Yuan et al., 2009; Gielen et al., 2009; Esmenjaud et al., 2019) and providing a key site for allosteric drug action. The NTDs also interact as dimers, but as the alternate dimers compared to the ABD (Karakas and Furukawa, 2014) and so complete a beautiful pseudo-symmetry with the potential for allosteric communication across the extracellular regions of the receptor and providing multiple regions of within-subunit and between-subunit interaction that form prime targets for the binding of allosteric modulators.

It is usually assumed that normal NMDA receptor activation requires all four subunits to be agonist occupied (Clements and Westbrook, 1991), while for AMPA receptors (Rosenmund et al., 1998) channel openings can occur when not all agonist binding sites are occupied. Agonist binding to the bi-lobed agonist binding domain of each receptor subunit initiates a conformational change (a ‘pre-gating’ movement) that propagates through the linker region of each subunit to the transmembrane domains to facilitate channel opening.

### 1.2 NMDA receptor activation mechanisms: how does an allosteric antagonist work?

**Fig. 2:**
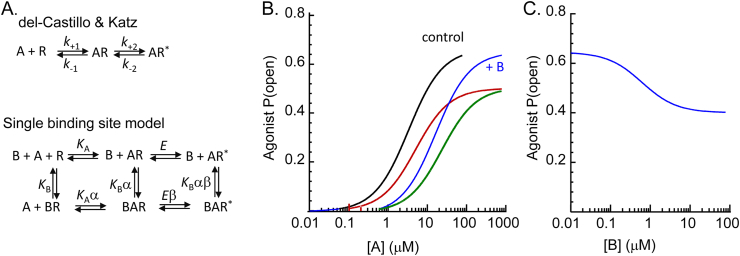
Single binding site models. In **A**, extension of the del-Castillo & Katz model to include binding of an allosteric antagonist is illustrated with allosteric coupling factors α, to describe the change in agonist, *K*_A_, or antagonist, *K*_B_, affinity following ligand binding or β, to describe a change in agonist efficacy, *E*. With this definition, when α > 1, affinity is decreased or when β > 1 efficacy is decreased. In **B**, is shown example agonist dose-response curves (*K*_A_ = 10 μM, *E* = 2) in control (black, *EC*_50_ = 3.3 mM, *P*_o(max)_ = 0.67) and in the presence of 10 μM allosteric antagonist for the condition where the allosteric modulator changes agonist affinity (α = 5, blue, *EC*_50_ = 16 μM, *P*_o(max)_ = 0.67) or efficacy (β = 2, red, *EC*_50_ = 5 μM, P_o(max)_ = 0.5) or both (green, *EC*_50_ = 23.5 μM, *P*_o(max)_ = 0.5). Panel **C**, shows the antagonist inhibition curve in the presence of [A] = 100 μM. The maximum inhibition is at *P*_open_ = 0.4. Simulations made using *Scalcs* from the DCprogs software suite (https://www.ucl.ac.uk/Pharmacology/dcpr95.html)(Colquhoun Hawkes, 1995). (For interpretation of the references to colour in this figure legend, the reader is referred to the Web version of this article.)

The del-Castillo & Katz (1957) model of receptor activation (Fig. 2a) provides a conceptual framework to understand activation of ion channel receptors and possible mechanisms of action of allosteric modulators. This model distinguished beautifully between the binding of the agonist (A) to the receptor and subsequent receptor activation. Agonist affinity is described by the dissociation equilibrium constant, *K*_A_, and subsequent conformational changes in the receptor protein are represented by the efficacy of receptor activation, *E* (where *K*_A_ = *k*_-1_/*k*_*+*1_ and *E* = *k*_-2_/*k*_+2_). In reality, several changes in protein conformation are represented in this model by the efficacy step, *E* (Banke and Traynelis, 2003; Schorge et al., 2005; Erreger et al., 2005; Auerbach and Zhou, 2005). In the del-Castillo & Katz model it is clear that the proportion of receptors in the active state, *P*_AR*_ depends on both *K*_A_ and *E* while, perhaps less obvious, the half-maximum concentration on the agonist dose-response curve, also depends on both *K*_A_ and *E:* [A]_50_ = *K*_A_/(1 + *E*) (Colquhoun, 1998). Also less obvious, In addition, the synaptic response time course also depends on both agonist affinity and pre-gating movements in the protein (Lester and Jahr, 1992; Wyllie et al., 1998; Popescu et al., 2004; Erreger et al., 2005). An example of this is in the analysis of models used to describe NMDA receptor data by Lester and Jahr (1992). They investigated the effect of including a long-lived closed state (a ‘desensitized’ state) in their model of the receptor activation. Including this state in the model means that on average the agonist stays bound to the receptor for longer during each activation giving an apparent increase in agonist affinity reflected in the [A]50 value of the agonist dose-response curve. Increasing the time that the agonist stays bound to the receptor also prolongs the predicted synaptic current, providing a good explanation for the slow kinetics of the NMDA receptor mediated synaptic current.

With an allosteric antagonist, drug binding to the receptor can occur whether the agonist binding site is occupied or vacant, and whether the ion channel is closed or open. Thus, taking antagonist (B) binding into account creates cycles in the mechanism (Fig. 2b). Both agonist affinity and/or the efficacy of receptor activation may be altered to a greater or lesser extent depending on whether the allosteric antagonist is bound. The degree of effect of antagonist binding on agonist binding (and *vice-versa*) are described here by the coupling constant α (allosteric constant), while the magnitude of effects on receptor efficacy are described by β. Note that in the del-Castillo & Katz model illustrated in Fig. 2, α and β are applied to the antagonist dissociation rate and so α, β > 1. An alternative formalism could apply the allosteric constant to the antagonist association rate, and so for inhibition α, β would be less than unity. Fig. 2b illustrates these two scenarios in terms of the shape of the agonist dose-response curve. The control dose-response curve is for an agonist with *EC*_50_ = 3.3 μM and a maximum channel open probability of *P*_o(max)_ = 0.67. In the presence of 10 μM allosteric antagonist (*K*_B_ = 0.1 μM) that decreases agonist affinity (α = 5), the dose-response curve is shifted to the right (*EC*_50_ = 16 μM) with no change in the maximum. In the presence of an allosteric antagonist that decreases efficacy (β = 2), the dose-response curve is shifted slightly to the right (*EC*_50_ = 5 μM) with a decrease in the maximum to *P*_o(max)_ = 0.5. The third example simulation shows the agonist dose-response curve in the presence of an allosteric antagonist that affects both agonist affinity (α = 5) and efficacy (β = 2). The dose-response curve is shifted to the right (*EC*_50_ = 23.5 μM) with a decrease in the maximum to *P*_open_ = 0.5. The inhibition curve for such an allosteric antagonist is illustrated in Fig. 2c. In the presence of a constant concentration of agonist ([A] = 100 μM), the curve begins at an antagonist concentration [B] = 10 nM and at a *P*_open_ = 0.64. With increasing [B], *P*_open_ decreases with an *IC*_50_ = 0.7 μM, reaching a maximum inhibition at *P*_open_ = 0.4. Although not considered in detail in this article, similar principles can be applied to modelling the action of a positive allosteric modulator (‘PAM’) drug, in that both agonist affinity and/or channel open probability may be altered by binding of the drug to the receptor.

The principle of microscopic reversibility applied to the cycles in the activation mechanism in Fig. 2A means that the product of the rates of the reactions going clockwise round a cycle must equal the product of the reactions going anti-clockwise. For a cycle with four equilibria, this means there is one equilibrium constant which depends on the other three. This can also be envisioned by considering that the free energy of formation of state ARB from R must be the same whether A or B bind first. In the reaction in Fig. 2A, the equilibrium constant for agonist binding, *K*_A_ is therefore multiplied by the factor α when the antagonist is already bound and likewise, if the agonist is bound first, then *K*_B_ is multiplied by α. This illustrates the reciprocal nature of allosteric effects: if agonist affinity is altered by the antagonist binding, then there must be a reciprocal change in antagonist affinity when the agonist binds. With this definition of α, when α > 1, affinity is decreased.

### 1.3 Biophysical evidence for triheteromeric GluN1/GluN2B/GluN2D receptors

**Fig. 3:**
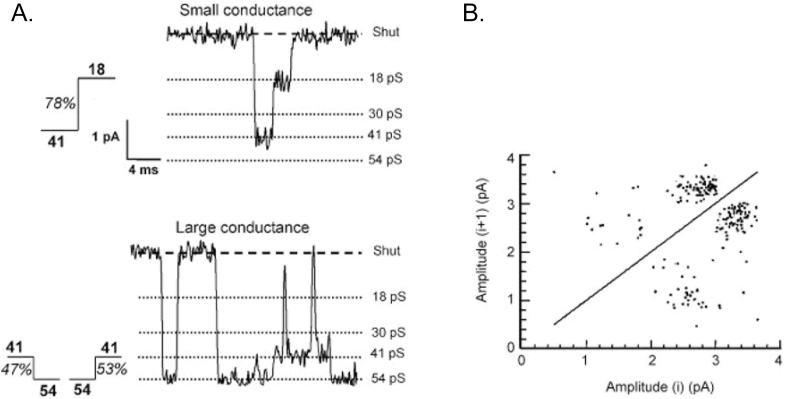
Direct transitions between conductance levels in the single channel current characteristic of GluN2D subunit-containing receptors. A, examples of direct transitions between 18 pS and 41 pS (small conductance) and 41 pS and 54 pS (high conductance), with percentage occurrence observed in this patch indicated for each transition. B, plot of channel amplitudes before and after direct transitions from the same patch illustrated in A. Each point on the graph represents a single direct transition. The density of points illustrates that direct transitions between 41 pS and 54 pS occur with equal frequency, while transitions between 18 pS and 41 pS are asymmetric, occurring more frequently from 41 pS to 18 pS than from 18 pS to 41 pS. *Adapted from* (Jones and Gibb, 2005)*.*

Early functional evidence for triheteromeric NMDA receptors in central neurons containing GluN2B and GluN2D subunits came from a combination of pharmacology and biophysics. In neonatal hippocampal neurons (Pina-Crespo and Gibb, 2002), cerebellar Golgi cells (Brickley et al., 2003) and substantia nigra dopaminergic neurons (Jones and Gibb, 2005). NMDA activates single channel currents with properties corresponding to both GluN2B and GluN2D subunit-containing diheteromeric receptors. In particular, an asymmetry in the frequency of direct transitions (Fig. 3) in the single channel current between low-conductance (∼18 pS) and intermediate conductance (∼40 pS) openings was observed (in substantia nigra: 18–41 pS; 36%, 41–18 pS; 64%) (Fig. 3a). For each receptor subtype, the single channel conductance properties depend on all subunits that make up the receptor. Different combinations of subunits give rise to variations of channel properties with the asymmetry in frequency of direct transitions between conductance levels being a feature that is unique to GluN2D subunit-containing receptors (Wyllie et al., 1996). Crucially, in substantia nigra this asymmetry extended to direct transitions between low-conductance and high conductance openings (18–54 pS; 41%, 54–18 pS; 59%) (Fig. 3) suggesting that the substantia nigra receptors had channel properties of both GluN2B (54 pS conductance) and GluN2D (18 pS conductance) and was able to transiently switch between these. Because asymmetry of direct transitions from 18 pS to 54 pS was observed, this evidence implies that GluN2B and GluN2D subunits are present within the same receptor. Interestingly, the receptors in substantia nigra also have a reduced Mg^2+^ block sensitivity and a voltage-dependence (Huang and Gibb, 2014) intermediate between that expected for A/B receptors and that expected for C/D receptors. In each case, NMDA currents were also sensitive to ifenprodil (or CP-101,606) while in the Golgi cells, gene deletion of GluN2D was also found to alter the single channel properties. Thus in the absence of GluN2D expression, the GluN2D-type single channel properties disappeared, indicating that normally in Golgi cells, GluN2D subunits are part of the receptor.

### 1.4 Triheteromeric GluN1/GluN2B/GluN2D receptors mediate synaptic currents in subthalamic nucleus and substantia nigra of the basal ganglia and in hippocampal interneurons

**Fig. 4:**
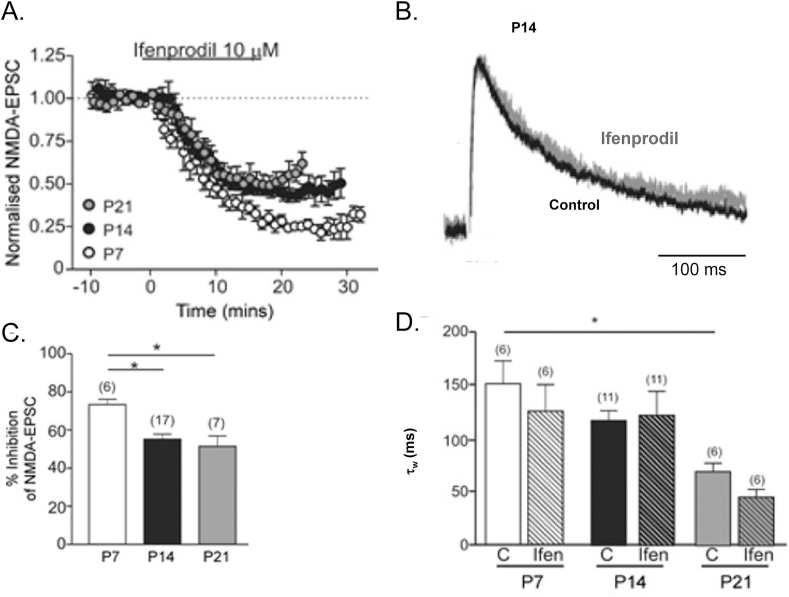
Ifenprodil block of synaptic NMDA receptors in substantia nigra dopaminergic neurons. Panel **A**, combined data of stimulus-evoked NMDA-EPSCs recorded over time during application of ifenprodil (10 μM) at three developmental stages (open circles, P7 days, n = 6; black circles, P14 days, n = 17; grey circles, P21 days, n = 7). In all three age groups ifenprodil caused a significant inhibition. Panel **B**, example recordings of NMDA-EPSCs at +40 mV from a SNc dopaminergic neurone in a slice from a rat aged P14 days. Traces are the average of 20 NMDA-EPSCs. ‘Control’ EPSC (black trace) was recorded in picrotoxin (10 μM), glycine (10 μM) and DNQX (10 μM) to block GABA_A_, Glycine and non-NMDA receptors. ‘Ifenprodil’ EPSC (grey trace) was recorded in the presence of 10 μM ifenprodil, and the two traces have been scaled to allow comparison of their decay. Panel **C**, bar graph comparing the mean inhibition (%) induced by 10 μM ifenprodil at P7 (measured 20–30 min post-drug), P14 and P21 (both measured 10–20 min post-drug). Significant difference detected with ANOVA; *P < 0.01, Bonferroni's multiple comparison post hoc test (‘n’ in parentheses). Panel **D**, Bar graph of the summary results shows the weighted decay time constant (τ_W_) of the NMDA-EPSC decay (weighted from a two-component exponential fit of the NMDA-EPSC at P7, P14 and P21 days) in control recordings and from recordings in the presence of 10 μM ifenprodil at each age. ANOVA revealed a significant difference (P < 0.05); post hoc tests revealed a significant difference between control τ_W_ at P7 and P21 (∗P < 0.05, Bonferroni's multiple comparison post hoc test; ‘n’ in parentheses). *Adapted from**Brothwell* et al. *(2008)**.*

In dopaminergic neurons of the substantia nigra (Brothwell et al., 2008), in subthalamic neurons (Swanger et al., 2015) and in hippocampal interneurons (Yi et al., 2019) the available evidence suggests that triheteromeric receptors containing GluN2B and GluN2D subunits are the predominant synaptic NMDA receptors. In these neurons the GluN2B selective antagonists ifenprodil (Fig. 4) or CP-101,606 were found to inhibit both whole-cell and synaptic currents to a maximum of about 55–65% inhibition (Brothwell et al., 2008; Suarez et al., 2010; Swanger et al., 2015; Yi et al., 2019). In contrast, diheteromeric GluN2B receptors are expected to be maximally inhibited by about 90% by ifenprodil, depending on GluN1 subunit splice variant. These data in themselves leave uncertain whether the neurons express a mixed population of GluN2B and GluN2D diheteromers, and/or also express triheteromeric N1/2B/2D receptors. However, both the pharmacology and the EPSC kinetics suggest the majority of receptors are triheteromers.

One key to unravelling the mysteries of triheteromeric receptors has come from use of molecular genetic approaches to engineer the assembly of receptors with defined subunit composition (Hansen et al., 2014; Stroebel et al., 2014). These advances followed on from an ingenious approach of Hatton and Paoletti (2005) to use mutations in the Mg^2+^ binding site to pharmacologically isolate recombinant triheteromeric receptors expressed in frog oocytes. Subsequently, using engineered expression of triheteromeric receptors with GluN2B and GluN2D within the same receptor, Yi et al. (2019), in a landmark paper, demonstrated conclusively that triheteromeric N1/2B/2D receptors are only partially inhibited by ifenprodil or CP-101,606 (to a maximum of 60–70%). This matches closely the inhibition of synaptic and whole-cell currents in subthalamic nucleus (Swanger et al., 2015) and substantia nigra (Fig. 4) (Brothwell et al., 2008; Suarez et al., 2010). Furthermore the kinetics of genetically engineered triheteromeric N1/2B/2D receptors are intermediate between those of GluN2B diheteromers and GluN2D diheteromers (Yi et al., 2019). Block of synaptic currents by ifenprodil or CP-101,606 does not change the EPSC current decay kinetics in substantia nigra (Brothwell et al., 2008), (Fig. 4), in subthalamic neurons (Swanger et al., 2015) or in hippocampal interneurons (Yi et al., 2019). If the synaptic currents were generated by a mixed population of GluN2B and GluN2D diheteromers, we would expect the residual current decay in the presence of ifenprodil would be slower. In fact the GluN2B selective drugs reduce the NMDA EPSC amplitude, but did not change the EPSC decay time course. The fact that the synaptic current kinetics did not significantly change in the presence of ifenprodil suggests the synaptic receptors are predominantly GluN1/GluN2B/GluN2D triheteromeric receptors*.* Similar results have been described for the action of the GluN2B antagonist CP-101,606 whereas the GluN2D selective antagonist, NAB-14, which is directed at the ‘slower’ of the two GluN2 subunits resulted in a speeding of the synaptic current decay (Yi et al., 2019). Interestingly there is an approximately 10% difference in the maximal effect of CP-101,606 and of Ifenprodil at GluN1/GluN2B/GluN2D triheteromeric receptors*.* (Yi et al., 2019).

### 1.5 A subunit-dependent model of allosteric antagonist action at a triheteromeric NMDA receptor

**Fig. 5:**
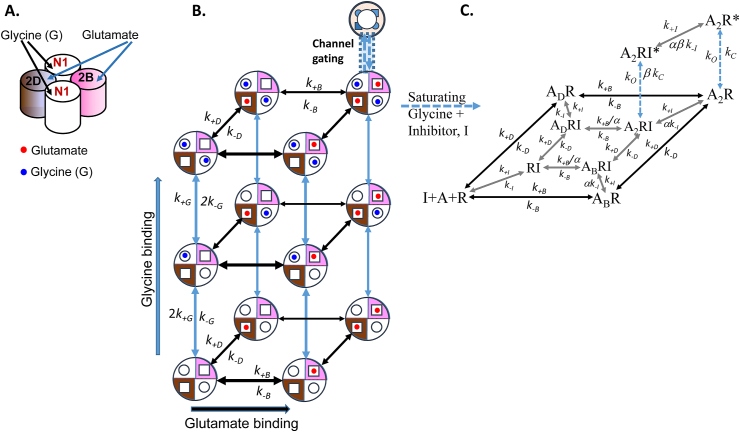
A. Cartoon representation of a GluN1/GluN2B/GluN2D triheteromeric receptor. B. A subunit-specific triheteromeric receptor activation model illustrating explicit glutamate and glycine binding to GluN1 and GluN2 subunits. C. is shown extension of this model to illustrate binding of an allosteric antagonist (I) to a single binding site on the GluN2B subunit. The model is shown assuming in the presence of a saturating concentration of glycine when the glycine binding steps are omitted.

Fig. 5 illustrates a triheteromeric subunit-dependent model of allosteric antagonist action at a GluN1/GluN2B/GluN2D triheteromeric receptor*.* The model in Fig. 5B illustrates glycine and glutamate binding to the respective GluN1 and GluN2 subunits, followed by a simple ‘efficacy’ step describing channel gating. For simplicity, the complications (and large increase in number of model states) of including possible ‘pre-gating’ conformational changes in the mechanism (Gibb et al., 2018) are omitted, although understanding the effects of allosteric antagonists on pre-gating is needed for a more detailed understanding of allosteric antagonist action.

A simplification made here (Fig. 5C) is to assume that the glycine sites are saturated during the experiment with a high concentration of glycine. Binding of the allosteric antagonist can occur whether or not the GluN2 subunits are occupied with a glutamate molecule.

The model shows:

i. Specific GluN2B and GluN2D subunit glutamate binding. Either subunit may bind glutamate first.
ii. A single binding site for the allosteric ligand, which in this example is assumed to be on the GluN2B subunit, and so a ‘coupling’ constant, α, (allosteric constant) is applied to the equilibrium constant (*K*_B_) for glutamate binding to the GluN2B subunit, and the coupling constant, β, to the ‘efficacy’ constant, *E*, for channel gating. The model does not include any change in glutamate binding to the GluN2D subunit following antagonist binding to the GluN2B subunit.

**Fig. 6:**
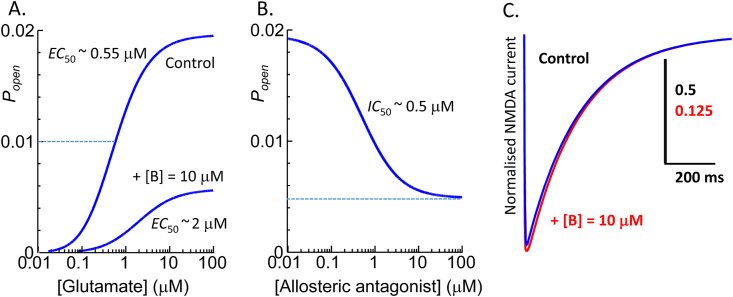
Simulation results from the GluN1/GluN2B/GluN2D triheteromeric receptor activation model shown in Fig. 5. A. Concentration-response curves for glutamate alone and in the presence of 10 μM allosteric antagonist. Model parameters were glutamate association rate constants for GluN2B and GluN2D subunits of *k*_+ B_ = *k*_+ D_ = 10^7^ M^−1^s^−1^ and dissociation rate constants *k-*_B_ = 4 s^−1^ = *k*_-D_ = 1 s^−1^. The rate constants for the allosteric antagonist (I) were *k*_+ I_ = 10^7^ M^−1^s^−1^ and *k-*_I_ = 1 s^−1^. Channel opening rate was 10 s^−1^ and channel closing rate, 500 s^−1^ with allosteric coupling factors α = 5 and β = 4. C. Simulated NMDA receptor synaptic currents activated by a 1 ms pulse of 1 mM glutamate in control and in the presence of allosteric antagonist (I) at 10 μM shown superimposed with amplitude normalised to allow comparison of the decay time course.

The simulation illustrates characteristics of a hypothetical allosteric antagonist that selectively binds to the GluN2B subunit, with properties chosen to be similar to real data described in Yi et al. (2019). However, note that the simulation does not include the possibility of changes in glycine binding or GluN1 subunit gating as part of the action of the allosteric antagonist. The simulation shows an allosteric antagonist (*K*_I=_100 nM, *IC*_50_ = 0.5 μM) with coupling constants α = 5 and β = 4 that at a concentration of 10 μM inhibits the maximum receptor response by about 75% (Fig. 6A and B) and shifts the glutamate dose-response curve with a dose ratio ∼ 4.

In order to simulate the synaptic current for this mechanism, rate constants were chosen that would give macroscopic kinetics, similar to those described by Yi et al. (2019) GluN1/GluN2B/GluN2D triheteromeric receptors. The rate constants for glutamate binding to the GluN2B and GluN2D subunits were respectively microscopic association rate constants *k*_+ B_ = *k*_+ D_ = 10^7^ M^−1^s^−1^ and dissociation rate constants *k-*_B_ = 4 s^−1^ = *k*_-D_ = 1 s^−1^ while the rate constants for the allosteric antagonist (I) were *k*_+ I_ = 10^7^ M^−1^s^−1^ and *k-*_I_ = 1 s^−1^. Channel opening rate was 10 s^−1^ and channel closing rate, 500 s^−1^ which in the absence of antagonist gives a maximum channel open probability, *P*_open_ = 0.02 (Fig. 6A). In this example, binding of the allosteric antagonist to the GluN2B subunit is assumed to cause a 5-fold decrease in glutamate binding rate, *k*_+ B_ (allosteric constant, α = 5). In addition, when glutamate is bound to the GluN2B subunit, the affinity of the allosteric antagonist is reduced by a 5-fold (α) increase in the inhibitor dissociation rate constant, *k-*_I_. With inhibitor bound to the GluN2B subunit, the channel closing rate (500 s^−1^) is increased to 2000 s^−1^ by the coupling factor of β = 4.

Simulation of a synaptic current initiated by a 1 ms pulse of 1 mM glutamate shows that pre-application of the antagonist at 10 μM inhibits the peak synaptic current by 75% but does not significantly change the synaptic current decay (Fig. 6; control decay tau = 203.6 ms, in presence of antagonist, tau = 204.2 ms). These simulations suggest a parsimonious explanation for the action of subunit-selective allosteric antagonists in that their action might be explained by a change in affinity and pre-gating within that subunit.

### 1.6 Concluding remarks

Compared to drugs acting at orthosteric sites on the receptor, allosterically acting drugs are potentially much more complicated to study, and even where the location of the binding site is known, it is difficult to make predictions about the drug action. The rich landscape of druggability and molecular selectivity promised by the potential variability of NMDA receptor allosteric sites comes at a price because we cannot extrapolate from knowledge of the location of the drug binding site to detailed predictions of drug action. Particularly with the NMDA receptor allosteric antagonists acting at triheteromeric receptors, the maximum drug effect is quite variable. In general with allosteric mechanisms, it could be said essentially ‘all bets are off’! The influence of any particular drug on agonist binding, pre-gating or channel opening and even single channel conductance cannot be assumed and instead must be measured to ascertain the mechanism of action of the drug. Despite the increased complications in understanding the mechanisms of action of allosteric antagonists, NMDA receptor pharmacology and drug discovery has seen rapid advances in recent years and allosteric antagonists now give tantalising hints at possibilities for new therapeutic agents in the future.

## Declaration of competing interest

I declare no Conflict of Interest in submission of this manuscript.
